# Academic Stress and Adolescents Mental Health: A Multilevel Structural Equation Modeling (MSEM) Study in Northwest of Iran

**DOI:** 10.34172/jrhs.2020.30

**Published:** 2020-10-31

**Authors:** Zahra Hosseinkhani, Hamid-Reza Hassanabadi, Mahboubeh Parsaeian, Mehrdad Karimi, Saharnaz Nedjat

**Affiliations:** ^1^Metabolic Diseases Research Center, Research Institute for Prevention of Non-Communicable Diseases, Qazvin University of Medical Sciences, Qazvin, Iran; ^2^Department of Educational Psychology, Kharazmi University, Tehran, Iran; ^3^Department of Epidemiology and Biostatistics, School of Public Health, Tehran University of Medical Sciences. Tehran, Iran; ^4^Department of Epidemiology and Biostatistics, School of Public Health, Knowledge Utilization Research center, Tehran University of Medical Sciences. Tehran, Iran

**Keywords:** Academic stress, Mental health, Self-concept, Adolescents, Iran

## Abstract

**Background:** We aimed to determine the relation of different sources of academic stress and adolescents´ mental health through mediator variables on the student and school levels.

**Study design:** A cross-sectional study.

**Methods:** Overall, 1724 students aged 12-19 yr were selected from 53 high schools in Qazvin City, northwest instead of central Iran through stratified cluster sampling. The sources of academic stress include family conditions, education system, future concerns, academic competitions, interaction with teachers, school disciplines, peer pressure, parental involvement, and financial problems. Academic self-efficacy and self-concept were the mediator constructs. The students and schools´ information were considered on levels 1 and 2, respectively. A Multilevel Structural Equation Modeling (MSEM) analysis was done.

**Results:** High value of academic stress was associated with reduction of mental health. On the student level, the academic stress caused by the families 0.31 (95% CI: 0.28, 0.34), peers 0.29 (95% CI: 0.27, 0.32), and the education system 0.21 (95% CI: 0.18, 0.24) had the highest impact on the adolescentsˊ mental health, respectively. There was a direct and indirect relation between academic stress and mental health through the self-concept. On the school level, only family conditions stress had a relation with mental health (*P*=0.015, b=1.08). Academic self-efficacy showed no significant relation in the model.

**Conclusion:** The stress from the family is the most important source of stress associated with adolescent mental health. Self-concept unlike academic self-efficacy had an important mediating role in the relation between different sources of academic stress and adolescents' mental health.

## Introduction

Adolescent mental health is linked to psychophysical and behavioral changes of adolescence ^1^. Now mental health disorders are considered newly emerging problems in the low and middle-income countries ^2^. Since adolescents have spent most of their time at schools, so academic stress is considered as one of the major risk factor to their mental health ^3^. In some communities, the only task of the adolescents is having excellent academic performance, so, the educational related issues can severely affect their health ^4^.

In the Iranian education system, the most important source of the academic stress is participating to university entrance national exam (Konkour) ^5^. In a qualitative study on 450 Iranian high school students, who were willing to enter university, the academic stress had been caused by different sources ^6^. The research on other societies has similarly unveiled the different sources of adolescence stress ^7-9^. Hence, determining the relation of different sources of academic stress with adolescents mental health is important, but it has been overlooked in studies and it is necessary to explore the role of personality traits and mediator variables in this process. In this regard, two important mediator constructs involved in adolescents mental health, are self-concept and self-efficacy. Self-efficacy is the individual´s belief in his abilities to succeed in the given situation ^10^.

One of these situations is education, associated with academic self-efficacy ^11,12^. Self-concept is the set of personal attitudes, feelings, and knowledge of one´s abilities and skills that can significantly improve people´s lives ^13^. Since, adolescents´ health in many societies is affected by the popular culture and the education system, so it is not possible to decide on the effect of the education systems of other societies based on the findings from the aforesaid research. Certainly, it is not possible to propose a single approach based on the results from the few studies ^14^. Considering the sociocultural issues of families and the education system of Iran, especially the university national entrance exam, it is necessary to investigate this issue. In this study, we explored the relation of different sources of academic stress and mental health of Iranian adolescents. Moreover, we investigated this issue on the student and school levels (level-1 and 2) based on the mediating role of two mediator variables; academic self-efficacy and self-concept.

 The following hypotheses were examined in this study:

First, following Suldo et al ^15^ and Jayanthi et al ^16^ we expected that higher mental health was associated with lower source-related academic stress. Second, following Sawatzky et al ^17^ and Wenz-Gross et al ^18^ and Garton et al ^19^ we expected that higher academic self-efficacy and self-concept scores would mediate the effect of high academic stress on mental health. Finally, following László et al ^20^ and Lee-Flynn et al ^21^ we expected that different sources of academic stress would directly and indirectly be associated with mental health via the self-concept and academic self-efficacy mediator variables on the student and school levels.

## Methods

 This cross-sectional study was done with the descriptive-analytical approach. Target population were high school students in Qazvin City, in the northwest of Iran. The participants were selected using the multistage sampling method. First, we coordinated with the Department of Education of Qazvin Province, northwest instead of central Iran and then prepared a list of the first and second period of high schools. Public, private, and special schools (with entrance exam) were classified in each periods of the high schools. Based on number of the students in each category, the number of the schools was determined proportional to the size. Next, 30 students were randomly selected based on their age groups (grade) from 53 school. The students and their parents signed the written informed consent. Next, trained interviewer explored the research objectives and methods to the students, and the students were asked to complete the questionnaires with self-administrated method without mentioning their names.

 The study protocol was approved by the Institutional Review Board of Tehran University of Medical Sciences (IR.TUMS.VCR.REC.1396.4610).

We used four questionnaires to collect data: The academic stress among adolescents was measured using Adolescents Academic Stress Questionnaire (AASQ) ^22^. This questionnaire consisted of 48 questions about the following nine domains: family conditions, education system, future concerns, interaction with teachers, academic competition, school disciplines, peer pressure, parental involvement, and financial problems. The questions are ranked based on the five-point Likert scale from never/very rarely to frequently/always. The scores of domains range from 48 to 240. High values indicate high academic stress. The internal consistency of this questionnaire was in the range between 0.58 and 0.85 according to Cronbach´s alpha coefficient and its repeatability with Intra Class Correlation (ICC) was 0.80 (95% CI: 0.66-0.90) ^22^.

Adolescents academic self-efficacy was assessed using Morgan and Jink´s Self Efficacy Scale (MJSES) This 30-item questionnaire covers three domains, talent, effort and context and the questions are ranked based on the four-point Likert scale from “really agree” to “really disagree”. The scores of domains range from 30 to 120 ^23^. Cronbach´s alpha in our sample was 0.74; its validity per dimension is available ^24^.

Adolescents' self-concept was assessed using Piers-Harris Children's Self-Concept Scale-Second Edition (PHCSCS-2), designed for the children aged between 7-18 years. The truncated version of this questionnaire consists of 30 questions covering the following domains: behaviors, mental state and cognition, physical appearance, anxiety, pro-social popularity, happiness, and satisfaction in the six-point Likert scale ranging from 30 to180 ^25^. Cronbach´s alpha of the Persian version of this questionnaire was 0.9 ^26^.

The mental health of the adolescents was assessed using the Strengths and Difficulties Questionnaire (SDQ). This questionnaire consisted of 25-item that covers five-domains. The questions are ranked based on the three-point Likert scale ranging from 0 to 50 ^27^. The valid and reliable Persian version of the questionnaire has been used in this study ^28^.

We analyzed three models to examine the research hypotheses. In the first hypothesis model, we analyzed the relation of different sources of academic stress with mental health through structural equation modeling (SEM). The SEM analysis is, in fact, a combination of confirmatory factor analysis and multiple regression techniques. It consists of two parts, measurement model and structural model. In the second hypothesis model, we introduced two mediator variables, namely academic self-efficacy and self-concept to the model, and analyzed the indirect relations between the variables. In the third hypothesis model, we analyzed the relation of different sources of academic stress with mental health on the student and school levels with the mediating role of the variables through MSEM method. MSEM is a method for simultaneously comparing the complicated relationships among the latent variables on different levels. This technique offers advantages of the SEM and multilevel models and is adequately flexible for assessing the fitness of models and computing level-2 outcomes ^29^. Moreover, we done primary analyses for the MSEM analysis. We calculated the mean, standard deviation, reliability (intra class correlation), and correlation of the variables. We considered schools as clusters, and analyzed the mean difference between the study variables per school to assess the necessity of the multilevel analysis. Estimations were made using the Maximum likelihood Ratio (MLR) method, and the missing data was in the range between 0.2% and 2.8%.

We imputed the missing data in SPSS using the EM algorithm method. We analyzed goodness of fit of the model using the Comparative Fit Index (CFI); Tucker-Lewis Index (TLI); Root Mean Square Error of Approximation (RMSEA); Root Mean Square Residual (RMSR) indices. We reported RMSR on student and school levels. As regards the CFI/TLI index, the minimum acceptable and good results were assumed to be 0.9 and 0.95, respectively. Concerning the RMSEA index, values below 0.06 and 0.08 were considered to be good and acceptable, respectively. In addition, the RMSR values below 0.05 were considered to be good ^30^. Finally, we did primary and MSEM analyses in SPSS ver.23 (Chicago, IL, USA) and Mplus version 7.4, respectively.

## Results

We enrolled 53 schools and 1740 students. The response rate was 96%. Mean ± SD age was 15 ± 1.7 ranging from 12 to 19 years. Overall, 864 (50.1%) participants were female and 860 (49.9%) were male. Besides, 899 (52.1%) participants were in the first period of high school, while 825 (47.9%) students were in second period. The mean of the academic stress was created by different sources was varied from 2.13 (peer pressure) to 3.93 (future concerns). There was also a significant difference in the mean of the sources of academic stress at schools (*P*<0.001). The ICC of different sources of the academic stress was also in the range between 0.44 (school disciplines) and 0.81 (family conditions). It indicated the considerable difference between the variables in the study groups. The mean, standard deviation, intra class correlation and correlation of the variables have been shown in Table 1. The factor loading for the observed variables was varied from 0.40 to 0.80.

**Table 1:** The mean, standard deviation, intraclass correlation and correlation of the source-related to academic stress

**Variables**	**Mean**	**SD**	**Intra class correlation**	**Inter item correlation matrix**
Family conditions	2.29	1.13	0.81 (0.79, 0.83)	0.39 (0.26, 0.52)
Education system	2.78	1.72	0.77 (0.74, 0.79)	0.42 (0.26, 0.68)
Future concerns	3.93	1.44	0.76 (0.74, 0.78)	0.53 (0.41, 0.76)
Interaction with teachers	3.00	2.66	0.71 (0.65, 0.76)	0.49 (0.42, 0.59)
Academic competitions	3.22	1.92	0.67 (0.62, 0.71)	0.43 (0.36, 0.54)
School disciplines	2.24	1.42	0.44 (0.18, 0.60)	0.32 (0.24, 0.42)
Peer pressure	2.13	1.44	0.64 (0.54, 0.71)	0.42 (0.29, 0.56)
parental involvement	2.72	1.80	0.69 (0.65, 0.71)	0.43 (0.34, 0.53)
Financial problems	2.90	2.00	0.74 (0.72, 0.76)	0.48 (0.33, 0.76)

**Figure 1:**
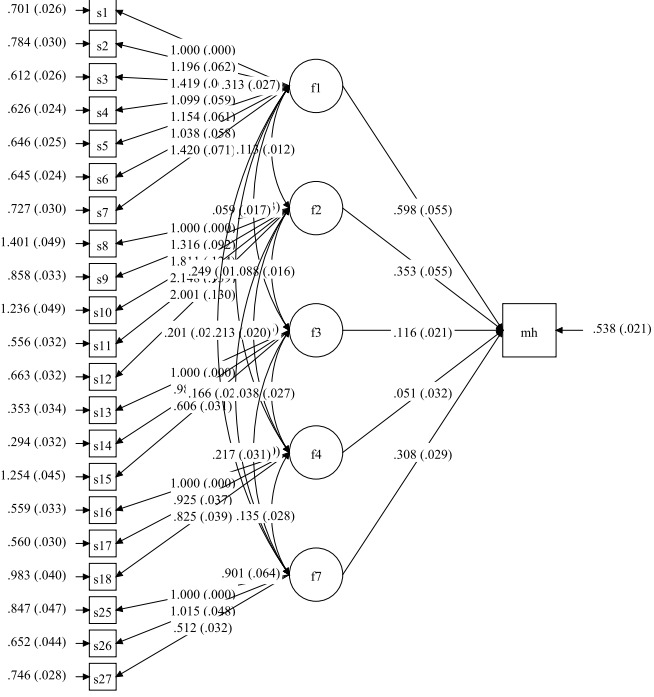
structural equation modeling with mental health as outcome, the source of academic stress, family conditions, education system, future concerns, teacher interaction and peer pressure as predictors. Standardized coefficients are shown. All paths are significant at *P*<0.05. f1= Family conditions, f2= Education system, f3= Future concerns, f4= teacher interactions, f7= Peer pressure, mh=Mental Health

The first hypothesis model addressed the relation of different sources of academic stress and mental health on the student level. The path analysis of the resulting model revealed the significant relation of mental health with the sources of academic stress which were family conditions, the education system, peer pressure, and future concerns (*P*<0.001) (Table 2). In addition, family conditions stress explained the mental health variance more than other sources 0.60 (95% CI: 0.54, 0.66) (Figure 1).

**Table 2:** Standardized Regression Coefficients (Standard Errors) Predicting Mental Health and Mediators variables

**Variables**	**Model 1**			**Model 2**			**Model 3**		
	**Standardized** **regression** **coefficients**	**SE**	***P *** **value**	**Standardized** **regression** **coefficients**	**Standard** **errors**	***P *** **value**	**Standardized** **regression** **coefficients**	**SE**	***P *** **value**
Path A									
Family conditions	0.33	0.03	0.001	0.07	0.03	0.001	0.07	0.02	0.005
Education system	0.18	0.03	0.001	0.10	0.02	0.001	0.13	0.02	0.001
Future concerns	0.12	0.02	0.001	0.08	0.02	0.001	0.08	0.02	0.001
Interaction with teachers	0.05	0.03		-0.04	0.03	0.201	-0.06	0.03	0.032
Peer pressure	0.31	0.03	0.001	0.15	0.02	0.001	0.13	0.02	0.001
Path B									
Family conditions	-	-	-	-0.44	0.02	0.001	-0.40	0.03	0.001
Education system	-	-	-	-0.12	0.02	0.001	-0.14	0.03	0.001
Future concerns	-	-	-	-0.04	0.02	0.003	-0.05	0.02	0.019
Interaction with teachers	-	-	-	-0.19	0.03	0.001	-0.14	0.04	0.001
Peer pressure	-	-	-	-0.26	0.03	0.001	-0.27	0.03	0.001
Path C		-	-						
Family conditions	-	-	-	0.27	0.03	0.001	-0.14	0.04	0.001
Education system	-	-	-	0.11	0.03	0.001	-0.05	0.04	0.239
Future concerns	-	-	-	-0.10	0.02	0.001	0.01	0.03	0.702
Interaction with teachers	-	-	-	0.32	0.03	0.001	-0.09	0.05	0.037
Peer pressure	-	-	-	0.14	0.03	0.001	-0.04	0.03	0.198
Path D		-	-						
Self-concept	-	-	-	-0.61	0.02	0.001	-0.60	0.03	0.001
Self-efficacy	-	-	-	0.01	0.02		-0.03	0.02	0.128
Between covariate variables									
Sex	-	-	-	-	-	-	-0.30	0.14	0.033
Educational period	-	-	-	-	-	-	-0.32	0.09	0.001
Goodness of fit									
χ2 (df)	910.84	844	0.001	1222	228	0.001	17928		0.001
RMSEA	0.046 (0.043,0.049)	0.050 (0.048, 0.053)	0.038
CFI/ TLI	0.941/0.930	0.934/0.920	0.929/0.915
SRMR (Within/Between)	0.040	0.040	0.037/0.226

Path A: outcome: mental health, Predictors: sources of academic stress Path B: outcome: self-concept. Predictors: sources of academic stress Path C: outcome: self-efficacy, Predictors: sources of academic stress Path D: outcome: mental health, Predictors: mediators variables

**Figure 2:**
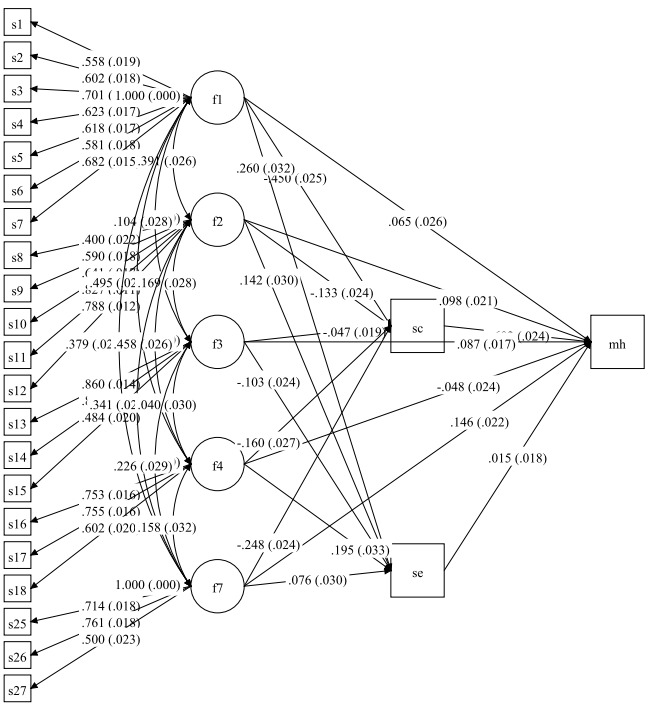
structural equation modeling with mental health as outcome, the source of academic stress, family conditions, education system, future concerns, teacher interaction and peer pressure as predictors, self-concept and self-efficacy as intermediate variables. Standardized coefficients are shown. All paths are significant at *P*<0.05. f1= Family conditions, f2= Education system, f3= Future concerns, f4= teacher interactions, f5= Peer pressure, mh=Mental Health, se=Self-Efficacy, sc=Self-Concept

In the second model, the academic self-efficacy and self-concept variables were also entered to the model as the mediator variables. In this model, mental health showed a significant relation with the sources of academic stress including; family conditions (*P*=0.001), education system, peer pressure, and future concerns (*P*<0.001). The sources of academic stress had a significant relation with self-concept and academic self-efficacy as the mediator variables (*P*<0.001). There was also a significant relation between adolescent self-concept and mental health (*P*<0.001), but the relation between academic self-efficacy and mental health was not significant (Table 2 and Figure 2).

In the third hypothesis model, the MSEM analyses were down on the student and school levels. On the student level, the academic stress caused by family conditions (*P*=0.005), education system (*P*<0.001), future concerns (*P*<0.001), interaction with teachers (*P*=0.032), and peer pressure (*P*<0.001) was significantly associated with the adolescent mental health. As regards the relation of the sources of academic stress with the mediator variables on this level, despite the significant relation of all sources of academic stress with self-concept (*P*<0.001), only the stresses caused by the family condition (*P*<0.001) and interactions with teachers (*P*=0.037) were significantly related to academic self-efficacy. In addition, the self-concept mediated the relation of the sources of academic stress with mental health (*P*<0.001), but academic self-efficacy did not have a significant role. On the school level, none of the sources of academic stress had a significant relation with mental health. Furthermore, sex (*P*=0.033) and high school educational periods (*P*<0.001) had a significant relation with mental health as level-2 variables. Table 2 presents the fitness index of all three models.

The results from the analysis of the overall, direct, and indirect effects of the sources of academic stress in the MSEM model are listed in Table 3. On the student level, the academic stress caused by different sources, directly and indirectly, influenced the mental health of the adolescents. On this level, the academic stress caused by family conditions (b=0.31, *P*<0.001), peers pressure (b=0.29, *P*<0.001), and education system (b=0.21, *P*<0.001) had the highest overall impact on the adolescent mental health, respectively. The regression coefficient for the relation between family conditions stress and mental health by the path of the self-concept mediator variable (b=0.24, *P*<0.001) was more than the direct path (b=0.07, *P*=0.005). Concerning the other sources of academic stress, the direct and indirect paths had almost equal shares (Table 2).

On the school level, none of the sources of academic stress had a significant relation with mental health via the direct, indirect, and overall paths except for the overall effect of family conditions academic stress on mental health (b=1.08l; *P*=0.015). The role of academic self-efficacy and self-concept (as the two mediator variables) in the relation between family conditions academic stress and mental health was not significant. However, the level 2 variables (i.e. sex (*P*<0.05) and high school educational periods (*P*<0.001) had a significant effect on mental health. In other words, the levels of mental health problems in the boy schools were lower than the girl schools. In the first period of the high school, the level of mental health problems was more than the second period. However, the relation of high school educational periods and sex with academic self-efficacy and self-concept was not significant. The values of three index of goodness of fit were acceptable or good; RMSEA were 0.046 (0.043, 0.049), 0.050 (0.048, 0.053) and 0.038, CFI/TLI were 0.941/0.930, 0.934/0.920, 0.929/0.915 and RMSR were 0.040, 0.040, 0.037/0.226 in three models, respectively.

**Table 3:** The Effects (standard errors) of variables in MSEM model

**Variables**	**Total effect**			**Direct effect**			**Total indirect effect**			**Indirect path self-efficacy effect**			**Indirect path self-concept effect**		
**Within level**	**Coefficients**	**SE**	***P*** ** value**	**Coefficients**	**SE**	***P*** ** value**	**Coefficients**	**SE**	***P*** ** value**	**Coefficients**	**SE**	***P*** ** value**	**Coefficients**	**SE**	***P*** ** value**
Family conditions	0.31	0.03	0.001	0.07	0.02	0.005	0.24	0.02	0.001	0.00	0.00	0.186	0.29	0.02	0.001
Education system	0.21	0.03	0.001	0.13	0.02	0.001	0.08	0.02	0.001	0.00	0.00	0.323	0.08	0.02	0.001
Future concerns	0.11	0.02	0.001	0.08	0.02	0.001	0.03	0.01	0.025	0.00	0.00	0.710	0.03	0.01	0.020
Interaction with teachers	0.03	0.04	0.391	-0.05	0.02	0.032	0.08	0.02	0.001	0.00	0.00	0.208	0.08	0.02	0.001
Peer pressure	0.29	0.03	0.001	0.13	0.02	0.001	0.14	0.02	0.001	0.00	0.00	0.364	0.16	0.02	0.001
Between level															
Family conditions	1.08	0.44	0.015	0.75	1.19	0.525	0.33	1.50	0.828	0.02	0.05	0.717	0.31	1.53	0.841
Education system	-0.42	0.25	0.085	-0.43	0.25	0.089	0.01	0.01	0.740	0.01	0.01	0.740	-	-	-
Future concerns	0.14	0.22	0.539	0.14	0.22	0.533	0.00	0.01	0.896	0.00	0.01	0.896	-	-	-
Interaction with teachers	0.01	0.66	0.986	0.00	0.39	0.994	0.01	0.29	0.962	0.00	0.02	0.983	0.01	0.27	0.957
Peer pressure	0.15	0.22	0.490	0.04	0.47	0.928	0.11	0.31	0.729	0.01	0.02	0.792	0.10	0.33	0.757
Between covariate															
Sex^*^	0.60	0.27	0.030												
Educational period^*^	-0.64	0.16	0.001												

* outcome

## Discussion

 The present study explored the relation of different sources of academic stress with adolescent mental health. The academic stress caused by family conditions, education system, future concerns, interaction with teachers, and peer pressure on the student level directly influences adolescent mental health. The effects of the academic stress caused by family conditions, peer pressure, and education system on adolescent mental health were more than the other sources of stress. On this level, the sources of academic stress were indirectly associated to adolescent mental health via self-concept (as a mediator variable). On the school level, all sources of academic stress except for family conditions did not have a significant relation with mental health via the direct and indirect paths. In addition, despite the negative relation of the sources of academic stress with academic self-efficacy in the present study, there was no significant relation between academic self-efficacy and mental health. Academic self-efficacy also did not mediate the relation between the sources of academic stress and mental health.

Academic stress has been introduced as a risk factor in different studies, but the risk level varies by student groups ^31,32^. The findings from this research revealed the significance of the role of family stress in mental health. About 29% of Chinese adolescents are suffering from family conflicts. Hence, given the important role of family coherence in the mental health of people, family history has always been emphasized along with the academic stress ^31^. Unlike the results from this study regarding academic self-efficacy, this variable was related to mental health, especially depression and anxiety, in different studies ^2,33^. For example, in Korea, adolescent self-efficacy was introduced as an important factor determining the adaptation of adolescents to academic stress ^4^.

The relationships of adolescents with their teachers and peers were also among the most important sources of academic stress, related to their mental health. Their relationships at school, in their families, and even outside schools are important, and teachers’ relationship with adolescents has always been considered an important determinant of the adolescents’ satisfaction ^4^. Besides, given the dependence of adolescents on their peers and schools, discrimination against them by their teachers and school principals is associated with their mental health. This discrimination can be a predictor of depression if adolescents are not supported, especially by their peers ^31,34^.

The results from this study concerning the mediating role of self-concept mirror the effect of social status and parents’ education on adolescents’ self-concept and their mental health^35^. Although adolescents interactions with their peers, parents behavior, and parents expectations of adolescent influence adolescents self-concept, adolescents self-concept also influences their interactions with teachers, learning activities, and adaptation to school. Therefore, it is necessary to take adolescent self-concept into account in the analysis of adolescents’ adaptation to their environment ^36,37^.

Adolescents’ sex, as a level-2 variable in the MSEM model, had a major role in the relation between the sources of academic stress and mental health. In different studies, the role of sex in the academic stress and mental health of the adolescents has been emphasized, as this variable can adjusted the effect of academic stress on academic self-efficacy ^31,33,38^. However, although the aforesaid sources of stress affect girls more than boys, there was no significant difference between their perceptions of academic stress as their perception was determined by their attitude ^39^.

In this research, the level of stress in the first grade of high school was higher, negatively influencing the students' mental health. In Iran, “educational guide” in nine-grade students is known as a major source of academic stress due to the importance of it in the future university field of adolescents. In addition, in other countries, academic stress varies by grade and age of students ^40^.

 There were limitations in this study. Since the questions were subjective, the responses to the questions also depended on the students´ perception of the questions. The questionnaires were completed at schools by the students with a self-administered approach, and despite the emphasis put by the researchers on the confidentiality of the questionnaires, students might have provided dishonest responses to the school-related questions. Besides, this research was a cross-sectional study, but it was not possible to determine the causal relation due to the impossibility of calculating the order of the causes and effects.

## Conclusion

 At the student level, academic stress from different sources affects adolescent mental health. Family condition academic stress affects adolescent mental health more than other sources, and only it can affect adolescents´ mental health in the school level. Finally, adolescents´ self-concept has a mediating role in the relation between different sources of academic stress and mental health, but no significant relation was observed with academic self-efficacy. Therefore, the policy makers in educational and health system can concentrate their health promotion programs on reducing the sources of academic stress or reduce their effect on adolescents´ mental health. Besides, they can empower adolescents to manage academic stress by improving their self-concept.

## Acknowledgements

 The authors thank all the students for participating in this study

## Conflict of interest

 None declare.

## Funding

 Tehran University of Medical Sciences funded this study.

## Highlights

- Family stress, peer pressure, and the current education system had the strongest relation with reduced mental health at the student level and this relationship was mediated by self-concept.
- At the school level, only family stress was associated with reduced mental health.
- The model varied by both school education periods and by sex.
- In all models, academic self-efficacy was not significant.
